# Assessment of Circulating lncRNA H19 in Ankylosing Spondylitis Patients and Its Correlation with Disease Activity

**DOI:** 10.3390/jpm13060914

**Published:** 2023-05-30

**Authors:** Marwa M. Esawy, Amany M. Ebaid, Amir Abd-elhameed, Felwa A. Thagfan, Murad A. Mubaraki, Ahmed S. Alazzouni, Mohamed A. Dkhil, Marwa A. Shabana

**Affiliations:** 1 Clinical Pathology Department, Faculty of Human Medicine, Zagazig University, Zagazig 44519, Egypt; 2 Rheumatology and Rehabilitation Department, Faculty of Human Medicine, Zagazig University, Zagazig 44519, Egypt; 3 Internal Medicine Department, Faculty of Human Medicine, Zagazig University, Zagazig 44519, Egypt; 4 Department of Biology, College of Science, Princess Nourah bint Abdulrahman University, Riyadh 11671, Saudi Arabia; 5 Clinical Laboratory Sciences Department, College of Applied Medical Sciences, King Saud University, Riyadh 12372, Saudi Arabia; 6 Department of Zoology and Entomology, Faculty of Science, Helwan University, Cairo 11795, Egypt; 7 Applied Science Research Center, Applied Science Private University, Amman 11931, Jordan

**Keywords:** lncRNA H19, ankylosing spondylitis, disease activity, predictor

## Abstract

Ankylosing spondylitis (AS) is a chronic inflammatory disease that results in severe pain and stiffness in the joints. The causes and pathophysiology of AS are still largely unknown. The lncRNA H19 plays key roles in the pathogenesis of AS by mediating inflammatory progression by acting in the axis of IL-17A/IL-23. The aims of this study were determining the role of lncRNA H19 in AS and assessing its clinical correlation. A case–control study was conducted and qRT-PCR was utilized to measure H19 expression. Comparing AS cases to healthy controls, it was found that H19 expression was significantly upregulated. For AS prediction, H19 demonstrated a 81.1% sensitivity, 100% specificity, and 90.6% diagnostic accuracy at a lncRNA H19 expression value of 1.41. lncRNA H19 had a significantly positive correlation with AS activity, MRI results, and inflammatory markers. lncRNA H19 seemed to be an independent predictor of AS (adjusted OR of 211 (95% CI: 4.7–939; *p* = 0.025)). After 3 months of clinical follow-up, seventeen patients (32.1%) showed minimal clinical improvement and fifteen patients (28.3%) showed major improvement. AS activity scores were significantly decreased in patients with high H19 expression. A significantly elevated lncRNA H19 expression was observed in AS cases compared with that in healthy controls. These results suggest that upregulation of lncRNA H19 expression may be involved in the pathogenesis of AS. The expression of the lncRNA H19 is related to the duration and activity of the disease. LncRNA H19 expression seems to be an independent predictor of AS.

## 1. Introduction

The sacroiliac and spine joints are the main targets of ankylosing spondylitis (AS), a chronic autoimmune-mediated inflammatory disease that results in severe pain and stiffness in these joints [1,2]. Additionally, ankylosis and spinal immobility develop in people with chronic AS. AS can considerably lower their quality of life [3,4].

Although there is no known etiology for AS, the disease’s molecular pathophysiology includes elements related to genetics, the immune system, microbes, and hormones [5]. HLA-B27 alone accounts for 20% of AS genetics because it is found in more than 80% of individuals who suffer from it [6]. The genetic test for HLA-B27 and non-specific acute phase reactants such as C-reactive protein (CRP) and erythrocyte sedimentation rate (ESR) are the biomarkers currently utilized in daily clinical practice for AS [7]. HLA-B27 was revealed to offer predictive data on phenotype and radiographic damage [8]. A reliable biomarker that can identify disease activity, spinal progression, and therapy response is one of the real needs in AS management [9].

Despite not being able to code for proteins, RNA transcripts that are categorized as long non-coding (lnc) RNAs have more than 200 nucleotides and can post-transcriptionally affect the expression of particular genes. The primary way that lncRNAs regulate gene transcription and translation is through interactions with other molecules, either directly or indirectly. These interactions can take place at different regulatory levels as well as in one simple level in the regulatory pathways [10]. Numerous biological processes, including cell division, apoptosis, and the release of pro-inflammatory cytokines, may be significantly regulated by lncRNAs, according to studies [11,12,13]. LncRNAs contribute to the development of orthopedic conditions [14,15,16,17,18]. Growing evidence indicates that lncRNAs are functionally involved in the pathophysiology of scoliosis [14]. Deregulated expressions of lncRNAs may have a role in the occurrence of osteosarcoma, osteoporosis, and intervertebral disc degeneration [15]. Altered expressions of lncRNAs have been implicated in the pathogenesis of different forms of arthritis, including rheumatoid arthritis and osteoarthritis [16]. Uncertainty still exists regarding the specific roles that lncRNAs play in the etiology of AS. According to the competing endogenous RNA (ceRNA) theory, which was recently proposed, lncRNAs and other RNA molecules with miRNA response regions may compete with one another for binding to a single miRNA, controlling miRNA-mediated gene silence [18].

LncRNA H19 is highly expressed in the majority of early embryonic tissues and the placenta, which drastically declines after birth with the exception of a few adult tissues such as skeletal muscle, cartilage, and cardiac muscle. lncRNA H19 RNA controls RNA or ribosomes and is primarily found in the cytoplasm. LncRNAH19 is essential for controlling a variety of cellular functions. As a competitive endogenous RNA, lncRNA H19 first controls mRNA expression by “sponging” target miRNA. Second, via interacting with numerous proteins, lncRNA H19 is involved in controlling many activities in diverse types of cells. Thirdly, by enlisting histone-methylation-related epigenetic regulatory mechanisms, H19 can take part in epigenetic regulation to control gene expression [19]. The interleukin-17A/interleukin-23 signaling pathway and the lncRNA H19 play key roles in the pathogenesis of AS, according to a recent study that found that AS cases had considerably higher levels of the lncRNA H19 than healthy controls did [20]. Determining the role of lncRNA H19 in AS and assessing its clinical correlation with activity, duration, therapy response, and radiological spinal involvement were the aims of this study.

## 2. Subjects and Methods

### 2.1. Study Design and Participants

After receiving approval from the Institutional Review Board of the Faculty of Medicine at Zagazig University (IRB No.: 9867), this study was carried out in the follow-up unit of the Rheumatology and Rehabilitation Department. The Clinical Pathology Department carried out the sample analysis. This study was carried out from July 2022 until January 2023. By signing a written informed consent form, each participant in the study indicated their agreement to take part. The rules outlined in the World Medical Association’s Code of Ethics were followed by this study methodology (Declaration of Helsinki).

The number of AS patients needed for this case–control research was estimated at 53. In addition, 53 healthy volunteers of similar age and sex who had no family history or obvious signs of an autoimmune disease served as controls in this study. Epi Software version 6 (Atlanta, GA, USA) computed the sample size at a 95% confidence interval (CI), assuming a mean difference expression of 0.6 and standard deviations of controls and cases (0.4 and 1.5, respectively). We chose patients using a simple random selection technique.

The patients were diagnosed according to the most commonly used criteria (2016 New York revised criteria) that are applied for too early a diagnosis of AS. The 1984 modified New York criteria were updated to exclude limited chest expansion and to add pelvic MRI, HLA-B27 positivity, positive family history of AS, enthesitis/arthritis, and a positive sacral push test. According to the New York revised clinical criteria 2016, a patient is diagnosed with AS if they receive at least 5 out of 13 points [21]. The following individuals were excluded from the study: those who declined to provide written consent; those who have significant comorbidities (such as cancer, a mental disorder, or other inflammatory or autoimmune diseases); and pregnant patients.

### 2.2. Clinical Examination

A comprehensive medical history and physical examination were performed on all patients. Using the AS Disease Activity Score (ASDAS), patients with AS were evaluated for disease activity. Patients were categorized into 4 disease activity statuses, including inactive disease (<1.3), moderate disease activity (<2.1), high disease activity (≤3.5), and very high disease activity (>3.5) [22]. Based on the ASDAS response criteria, two improvement scores were calculated: minimal clinically important improvement (MCII) (−1.1) and major improvement (−2.0) [23]. The degree of functional limitation in patients with AS was measured using the Bath AS Functional Index (BASFI) [24] and the Bath Ankylosing Spondylitis Disease Activity Index (BASDAI) [25]. The Maastricht Ankylosing Spondylitis Enthesitis Score (MASES), which scores 13 sites, was used to compute the overall sum of all site scores [26].

### 2.3. Radiological Evaluation

The degree of sacroiliac joint involvement in AS patients was assessed using magnetic resonance imaging. Data were documented to determine whether lesions in the sacroiliac joint quadrants (erosions, fat metaplasia), or sacroiliac joint halves (backfill, ankylosis), were present or absent. Each slice has a score between 0 and 8 for erosion and 0 to 8 for fat metaplasia (total score 0–40). Backfill and ankylosis were scored on a scale of 0 to 4 (total score 0–20) [27].

### 2.4. Samples

All participants provided four milliliters of whole blood, which was collected in two EDTA tubes. One tube was centrifuged for 10 min at 4 °C at a speed of 1200× *g*. After that, plasma samples were put into tubes free of RNase and DNase. The other tube was utilized to extract DNA to evaluate HLA-B27. All participants gave two milliliters of their whole blood, which was drawn into a plain tube. To separate the serum, the tube was centrifuged for 10 min at 1200× *g* speed. A total of 1.6 mL of whole blood was drawn and placed in an ESR tube. In this study, vacutainers from Becton Dickinson Company (Franklin Lakes, NJ, USA) were used.

### 2.5. Methods

The Vision B automated analyzer was used to determine the ESR (YHLO Biotech Co., Shenzhen, China). On the Cobas 8000/c702 analyzer, serum was utilized to assess the levels of CRP (Roche Diagnostic, Mannheim, Germany).

DNA extraction, PCR amplification, hybridization of the amplification products to a test strip containing allele-specific oligonucleotide probes, and enzymatic colorimetric detection are all steps in the HLA-B27 detection process from EDTA whole blood. The HLA-B27 StripAssay kit was used in accordance with the manufacturer’s instructions for each step (ViennaLab Diagnostics GmbH, Vienna, Austria).

The expression of LncRNA H19 in plasma was evaluated using quantitative real-time PCR. Using the miRNeasy Serum/Plasma Kit from QIAGEN, GmbH, Hilden, Germany, total RNA was extracted from plasma as directed by the manufacturer. Thermo Scientific’s NanoDrop-2000 spectrophotometer (USA) was used to measure the concentration and purity of the extracted RNA. Then, total RNA was reverse-transcribed into cDNA using the miScript RT II kit (QIAGEN GmbH, Hilden, Germany).

The cDNA templates were subjected to real-time RT-PCR using the miScript SYBR Green PCR kit in line with the manufacturer’s recommendations. The StepOneTM System real-time PCR instrument (Applied Biosystems, Foster City, CA, USA) was used to determine the level of lncRNA H19 expression. After a 15 min initial denaturation period at 95 °C, there were 40 cycles that each lasted 10 s at 94 °C, 30 s at 60 °C, and finally 1 min at 72 °C. A melting curve was analyzed to determine the specific amplification. β-actin expression was used to normalize the level of lncRNA H19 expression.

β-actin (forward: 5′-CTACCTCATGAAGATCCTCACC-3′, reverse: 5′-AGTTGAAGGTAGTTTCGTGGAT-3′) and lncRNA H19 (forward: 5′-TGCTGCACTTTACAACCACTG-3′, reverse: 5′-ATGGTGTCTTTGATGTTGGGC-3′) primers were used. 

Calculations were made to determine the lncRNA H19’s relative expression level by the 2^−ΔΔCT^ method [28].

### 2.6. Follow-Up

After 3 months of treatment with biological therapy, AS patients were re-evaluated. AS scores were re-estimated.

### 2.7. Statistical Analysis

All data were tabulated and statistically evaluated using SPSS 26.0 for Windows (SPSS Inc., Chicago, IL, USA). The Shapiro–Wilk test was used to determine whether the data were normal. Quantitative variables were displayed as a median and range, whereas categorical data were displayed as absolute values and percentages. The Mann–Whitney test and the Wilcoxon signed-rank test were applied to quantitative factors for unrelated and linked variables, respectively. The chi-square test was employed for comparing the categorical variables. The ability of the marker to predict was assessed using a receiver operating characteristic (ROC) curve analysis. The area under the ROC curve (AUC) and its 95% CI were used to assess performance. The Spearman correlation test was used to assess the relationship between the various study variables. By calculating the odds ratio (OR) and its 95% CI, the independent predictive indicators were found using logistic regression analysis. A *p*-value of 0.05 or less signifies statistical significance.

## 3. Results

**Figure 1:**
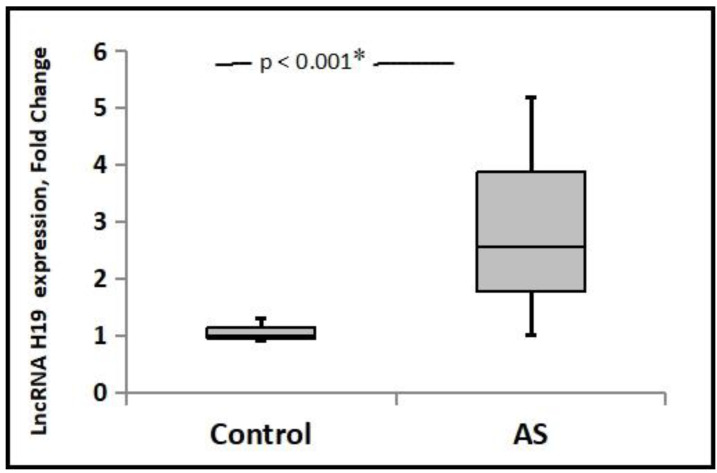
Expression levels of lncRNA H19 in AS patients and healthy controls *: Significant.

The controls and patients were matched in terms of age, sex, and smoking. Inflammatory indicators, including ESR and CRP, were significantly higher in AS patients. Table 1 displays the clinical and laboratory data for patients with AS. Figure 1 shows the expression of lncRNA H19 in AS patients and healthy controls. Comparing AS cases to healthy controls, it was found that H19 expression was significantly upregulated (*p* < 0.001). In AS patients, the median levels of lncRNA H19 expression were 2.55-fold changes (range: [1–5.2]). In AS patients with positive HLA-B27 compared to negative patients, the expression of the lncRNA H19 was higher (3.2 [1–5.2] and 1.02 [0.9–3.9], respectively) (*p* < 0.001). In AS patients, the frequencies of uveitis and secondary IBD were 7.5% and 13.2%, respectively. Between AS patients with and without uveitis, there was a non-significant difference in the expression of the lncRNA H19 (2.9 [2.3–5.2] and 2.5 [1–5.2], respectively; *p* = 0.41). In contrast to individuals without IBD, AS patients with IBD had higher levels of lncRNA H19 expression (2.5 [1–5.2] and 4.1 [1.5–4.7], respectively) (*p* = 0.01).

**Figure 2:**
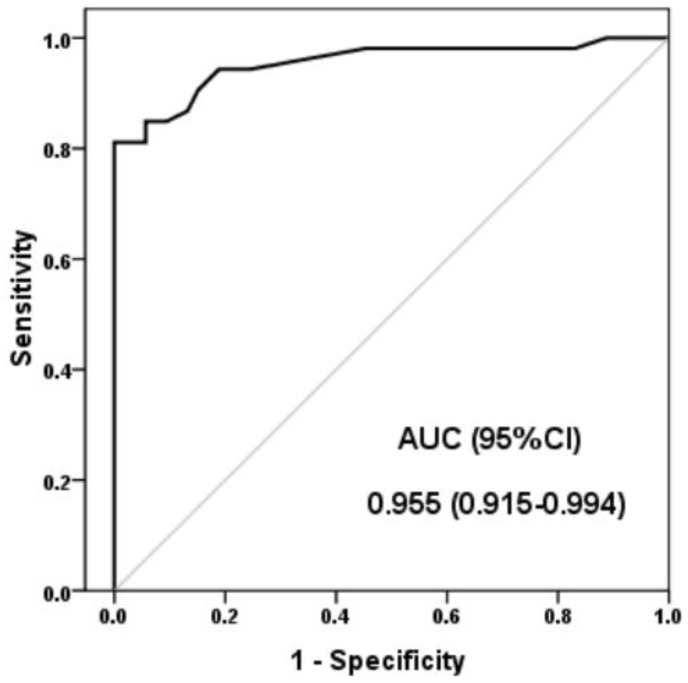
Receiver operating characteristic curves of lncRNA H19 expression for the diagnosis of AS. AUC: Area under curve; CI: Confidence interval.

A ROC curve analysis was used to assess the lncRNA H19 expression diagnostic performance values for AS. Figure 2 shows the ROC curve for lncRNA H19 as a predictive marker for AS. H19 demonstrated a 81.1% sensitivity, 100% specificity, 100% positive predictive value, 84.1% negative predictive value, and 90.6% diagnostic accuracy at a lncRNA H19 expression value of 1.41 (the maximum potential effectiveness of a biomarker (Youden’s index = 0.81)).

We investigated the associations between lncRNA H19 levels and the clinical features of AS patients (Table 2). LncRNA H19 and disease duration showed a positive correlation. Additionally, the correlation’s findings showed a significantly positive association between the activity of the disease and the levels of LncRNA H19. The levels of lncRNA H19 were positively correlated with the MRI results of erosion, ankylosing, and backfill (r = 0.34, r = 0.32, and r = 0.36, respectively) (*p* < 0.05). Inflammatory indicators (ESR and CRP) and lncRNA H19 were shown to be positively correlated (*p* < 0.001).

In the univariate analysis for AS prediction, lncRNA H19 expression was associated with an OR of 688 (95% CI: 34–1380). In addition, HLA-B27, ESR, and CRP levels were associated with AS. Other variables showed no correlations (Table 3). In the multivariate analysis including the variables presented in Table 3, lncRNA H19 expression and CRP are still significantly associated with AS. Both seem to be independent predictors of AS. The lncRNA H19 expression showed an adjusted OR of 211 (95% CI: 4.7–939).

**Figure 3:**
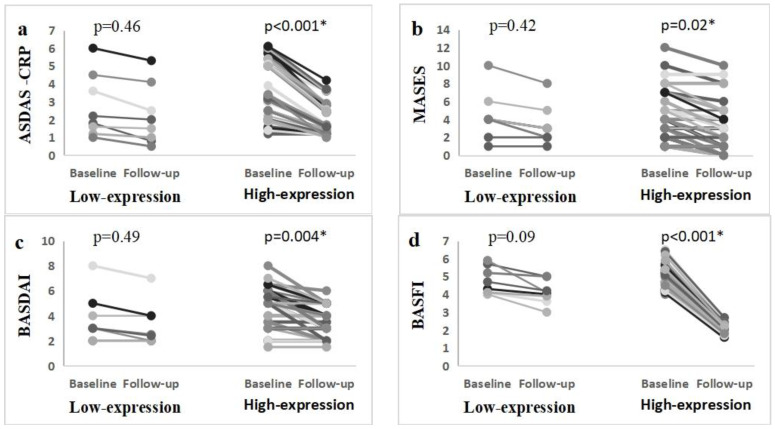
Changes in AS scores after 3 months. (**a**) ASDAS-CRP, (**b**) MASES, (**c**) BASDAI, (**d**) BASFI. Patients were classified regarding their H19 expression status. *: Significant by the Wilcoxon Signed-Rank Test.

After 3 months of clinical follow-up, patients were reevaluated. Seventeen patients (32.1%) showed minimal clinical improvement, and fifteen patients (28.3%) showed major improvement. BASDIA, BASFI, MASES, and ASDAS-CRP were significantly decreased in patients with high H19 expression, but those with low H19 expression levels showed an insignificant reduction in AS scores (Figure 3). 

## 4. Discussion

The causes and pathophysiology of AS are still largely unknown, leaving much to be discovered. The most recent research is still uncovering numerous chemicals and pathways that may play a role in the development of AS. Additionally, many AS patients continue to be dissatisfied despite recent advances in the drug sector (e.g., anti-TNF biologics, IL-17 inhibitors) [29]. Finding effective compounds that can be targeted to help with the management of this potentially crippling condition is imperative. Similar to mRNA, lncRNA is also transcribed and processed; however, it is unable to encode functional proteins. Nevertheless, they can influence the post-transcriptional expression of genes [30].

Early research by Xie et al. [31] showed the remarkable ability of AS mesenchymal stem cells (ASMSCs) to differentiate into osteoblasts and used microarrays to determine the lncRNA regulation. When comparing ASMSC to healthy donors, they found 520 lncRNAs that were differently expressed. Although it has recently been demonstrated that the expression of 159 lncRNAs can differentiate AS patients from controls, these differentially expressed genes could be implicated in the development of AS and had the ability of being markers of AS [32]. One of the lncRNAs that was shown to be abnormally overexpressed in AS patients as compared to healthy controls and was associated with AS susceptibility is lncRNA H19 [20]. In this study, we aimed to find a more relevant connection between H19 and AS disease activity parameters and, moreover, to explore a connection with radiological affection.

The HLA B27 antigen, critical in the pathogenesis of AS, is present in 80–90% of AS patients worldwide. In the current study, the HLA B27 antigen was found in only 60.4% of the patient group. This finding is in agreement with Abdelrahman et al. [33] who revealed that the prevalence of HLA-B27 among AS patients in the Arab world ranges from 56 to 84%, which is generally lower than the global number. These countries include Iraq (84%), the United Arab Emirates (56%), Saudi Arabia (67%), Egypt (58.6%), Syria (60%), and Iran (73.4%). 

In the current study, we found significantly upregulated H19 expression in AS cases compared to healthy controls by more than twofold; this finding is in line with the previous report that revealed that H19 was among the lncRNAs found to be upregulated in AS [29]. The more detailed observation we discovered is that lncRNA H19 is even more upregulated in patients with both AS and secondary IBD than in patients with only AS, supporting the finding that lncRNA H19 may play a role in compromising the intestinal epithelial barrier function [34]. The overexpression of H19 caused the expression of the Vitamin D Receptor (VDR) to be greatly reduced, which in turn caused permeability to increase and the expression of the two primary junction proteins (ZO-1 and occludin) to be lowered [35]. In addition, E-cadherin and ZO-1 are downregulated as a result of miRNA-675, and H19 acts as a precursor for miRNA-675 [36].

Regarding the correlations between lncRNA H19 levels and the clinical characteristics of AS patients, we found a statistically significant relationship between the disease duration and the level of H19 expression. Additionally, there was a significant positive correlation between LncRNA H19 levels and disease activity scores as well as between LncRNA H19 and inflammatory markers (ESR and CRP). These results support the recently proposed theory that LncRNA H19 plays a significant role in the pathogenesis of AS. It has been established that H19 acts by interacting with miR-675-5p and miR-22-5p, which in turn control the expression of VDR, IL17A, and IL23. The expression of inflammatory cytokines, including IL17A and IL23, was reduced because of H19 knockdown, which further clarifies these effects and explains the positive association between disease activity (as measured by ASDAS) and H19 levels. Studies on other cytokine-related disorders, including RA and OA, may make the posttranscriptional regulatory function of H19, which suggests that it is a critical molecular mediator in the development of inflammatory diseases, more obvious [37,38]. When compared to controls, the expression of H19 was higher in the synovial tissue of RA patients. Patients with OA had higher levels of LncRNA H19 expression in their peripheral blood, and it was also observed that the severity of damage was linked with it [39].

The ability of inflammatory lesions to remodel through a process known as fatty degeneration, which subsequently transforms into sites of new bone formation, has been well supported by MRI scans [40]. We found a highly significant correlation between the level of H19 expression in AS patients and new bone formation, or ankylosis, on their MRIs. This correlation can be explained by the numerous mechanisms by which H19 influences the pathogenesis of bone formation in AS. First, it was determined that H19 improves bone production by promoting osteoblast differentiation [41]. Second, lncRNA H19 can stimulate osteoblast development by triggering the Wnt/b-catenin signal pathway. This is accomplished by functioning as a competitor to miR22 and miR141 [42]. Previous studies have demonstrated the connection between Wnt signaling and the development of new bones in AS [43,44]. They investigated the impact of inhibiting Dickkopf-1 (DKK1), a Wnt antagonist previously associated with bone loss. When DKK1 was blocked, the disease’s phenotype changed from joint damage to remodeling and ankylosis.

In clinical applications, markers are employed to distinguish between normal and abnormal biological processes and to determine how well therapeutic interventions work. Inflammatory markers are associated with the causes and effects of many inflammatory disorders, and they may be predictive of inflammatory diseases. In several disorders, the biomarker may be compromised. Consequently, the clinical manifestations must be considered with the marker data [45]. LncRNA H19 demonstrated a 81.1% sensitivity, 100% specificity, and 90.6% diagnostic accuracy for the prediction of AS. To examine lncRNA H19, laboratory, and demographic factors, we performed univariate and multivariate logistic regression analysis. lncRNA H19 was independently linked to AS and had the ability to predict AS. 

By monitoring the patient’s status, reevaluating their disease activity, and tracking their therapeutic response using improvement indices, we observed that patients undergoing biological therapy responded better if their initial lncRNA H19 expression levels were higher. H19 is overexpressed in AS patients and mediates inflammatory progression by acting in the axis of H19-miR22-5p-VDR-IL-17A/IL-23 and interacting with miRNA in the axis of H19-miR675-5p-VDR-IL-17A/IL-23 [20]. So, patients with high H19 expression exhibit a significant improvement with biological drugs (anti-interleukin 17A), which inhibit the end product of these axes. Patients with low lncRNA H19 expression did not improve after biological drugs, which suggests the presence of other molecular mechanisms for the inflammatory process of ankylosing spondylitis. This finding will require further studies to be evidenced.

We noted that the current study has a few limitations. First, the sample size was relatively small, which made it necessary to gather a larger sample from other locations and include participants of various races. Second, we did not evaluate how well the lncRNA H19 could distinguish AS from other rheumatic illnesses. Third, the expression level of lncRNA H19 was not re-assessed after the follow-up period.

## 5. Conclusions

A significantly elevated lncRNA H19 expression was observed in AS cases compared with that in healthy controls. These results suggest that the upregulation of lncRNA H19 expression may be involved in the pathogenesis of AS. The expression of the lncRNA H19 is related to the duration and activity of the disease. LncRNA H19 expression seems to be an independent predictor of AS. 

## Figures and Tables

**Table 1:** Subjects’ demographics and clinical and laboratory characteristics.

Parameters		AS Patients (No.: 53)	Controls (No.: 53)	*p*
Age		41 [30–65]	42 [33–57]	0.51
Sex (Male/Female)		35/18 (66/34)	33/20 (62.3/37.7)	0.41
Smoking		6 (11.3)	10 (18.9)	0.32
Duration (Years)		8 [2–20]		
Type:				
	Axial	53 (100)		
	Peripheral	4 (7.5)		
ASDAS-CRP:		3.3 [1–6.1]		
	Inactive	8 (15.1)		
	Moderate	7 (13.2)		
	High Very high	15 (28.3) 23 (43.4)		
Morning stiffness score		3 [0–10]		
Patient global assessment score		5 [0–10]		
MASES		4 [1–12]		
BASFI		4.9 [4–6.5]		
Fatigue Score		3.3 [1–8]		
BASDAI:		5 [1.5–8]		
	Inactive	27 (50.9)		
	Active	26 (49.1)		
Associated conditions:				
	Uveitis	4 (7.5)		
	Secondary IBD	7 (13.2)		
MRI scores				
	Erosion	4.2 [2–8.5]		
	Fatty metaplasia	6.5 [2.3–29.5]		
	Ankylosing	2.6 [0–9]		
	Backfill	2.6 [0–10.2]		
Biological Treatment				
	Anti-interleukin 17A	39 (73.6)		
	Anti-tumor necrosis factor	14 (26.4)		
Laboratory findings				
	ESR, mm/h	21 [5–44]	6.8 [2–16]	<0.001 *
	CRP, mg/L	25.6 [2.1–87.2]	2.5 [0.9–7]	<0.001 *
	HLA-B27	32 (60.4)	0 (0)	<0.001 *

Data are expressed as median [Min-Max] or number (%). No: Number; AS: Ankylosing spondylitis; ASDAS: ankylosing spondylitis disease activity score; BASFI: Bath AS Functional Index; MASES: Maastricht Ankylosing Spondylitis Enthesitis Score; BASDAI: Bath Ankylosing Spondylitis Disease Activity Index; IBD: Inflammatory bowel disease; MRI: Magnetic resonance imaging; ESR: Erythrocyte sedimentation rate; CRP: C-reactive protein; HLA: human leukocyte antigen. *: Significant.

**Table 2:** Correlation of LncRNA H19 expression levels with AS patients’ characteristics.

Parameters of PsA		LncRNA H19	
		r_s_	*P*
Age		0.12	0.36
Duration		0.29	0.033 *
ASDAS-CRP		0.4	0.003 *
Morning stiffness score		0.41	0.002 *
Patient global assessment score		0.33	0.017 *
MASES		0.37	0.006 *
BASFI		0.34	0.014 *
BASDAI		0.57	<0.001 *
Fatigue Score		0.33	0.015 *
MRI Finding			
	Erosion	0.34	0.012 *
	Fatty metaplasia	0.24	0.85
	Ankylosing	0.32	0.018 *
	Backfill	0.36	0.009 *
ESR		0.63	<0.001 *
CRP		0.59	<0.001 *

AS: Ankylosing spondylitis; ASDAS: ankylosing spondylitis disease activity score; BASFI: Bath AS Functional Index; MASES: Maastricht Ankylosing Spondylitis Enthesitis Score; BASDAI: Bath Ankylosing Spondylitis Disease Activity Index; MRI: Magnetic resonance imaging; ESR: Erythrocyte sedimentation rate; CRP: C-reactive protein. *: Significant.

**Table 3:** Logistic regression analysis of risk factors for AS.

Covariate	Univariate Analysis		Multivariate Analysis	
	OR (95%CI)	*p*-Value	AOR (95%CI)	*p*-Value
Age	1.02 (0.97–1.07)	0.41		
Sex (Male)	1.17 (0.53–2.6)	0.69		
Smoking	0.55 (0.18–1.64)	0.28		
HLA-B27	18.6 (5.8–59.4)	<0.001 *	0.43 (0–364)	0.81
CRP	1.83 (1.36–2.44)	<0.001 *	1.82 (1.18–2.8)	0.03 *
ESR	1.32 (1.19–1.47)	0.001 *	1.28 (0.95–1.73)	0.09
LncRNA H19	688 (34–1380)	0.004 *	211 (4.7–939)	0.025 *

OR: Odds ratio; AOR: Adjusted odds ratio; CI: Confidence interval; HLA: human leukocyte antigen, ESR: Erythrocyte sedimentation rate; CRP: C-reactive protein. *: Significant.

## Data Availability

All data not published within this article will be made available by request from any qualified investigator.
